# Exploring the link between pyrethroids exposure and dopaminergic degeneration through morphometric, immunofluorescence, and *in-silico* approaches: the therapeutic role of chitosan-encapsulated curcumin nanoparticles

**DOI:** 10.3389/fphar.2024.1388784

**Published:** 2024-05-01

**Authors:** Badriyah S. Alotaibi, Amany Abdel-Rahman Mohamed, Yasmina M. Abd-Elhakim, Ahmed E. Noreldin, Moustafa Elhamouly, Tarek Khamis, Ali H. El-Far, Manal E. Alosaimi, Naief Dahran, Leena S. Alqahtani, Mario Nicotra, Mohamed El-Gamal, Alessandro Di Cerbo

**Affiliations:** ^1^ Department of Pharmaceutical Sciences, College of Pharmacy, Princess Nourah bint Abdulrahman University, Riyadh, Saudi Arabia; ^2^ Department of Forensic Medicine and Toxicology, Faculty of Veterinary Medicine, Zagazig University, Zagazig, Egypt; ^3^ Department of Histology and Cytology, Faculty of Veterinary Medicine, Damanhour University, Damanhour, Egypt; ^4^ Cytology and Histology Department, Faculty of Veterinary Medicine, University of Sadat City, Sadat City, Egypt; ^5^ Department of Pharmacology, Faculty of Veterinary Medicine, Zagazig University, Zagazig, Egypt; ^6^ Laboratory of Biotechnology, Faculty of Veterinary Medicine, Zagazig University, Zagazig, Egypt; ^7^ Department of Biochemistry, Faculty of Veterinary Medicine, Damanhour University, Damanhour, Egypt; ^8^ Department of Basic Health Sciences, College of Medicine, Princess Nourah bint Abdulrahman University, Riyadh, Saudi Arabia; ^9^ Department of Anatomy, Faculty of Medicine, University of Jeddah, Jeddah, Saudi Arabia; ^10^ Department of Biochemistry, College of Science, University of Jeddah, Jeddah, Saudi Arabia; ^11^ School of Biosciences and Veterinary Medicine, University of Camerino, Matelica, Italy; ^12^ Forensic Medicine and Clinical Toxicology Department, Faculty of Medicine, Mansoura University, Mansoura, Egypt; ^13^ Department of Biological Sciences, Faculty of Science, New Mansoura University, New Mansoura City, Egypt

**Keywords:** curcumin-encapsulated chitosan nanoparticles, dopamine, fenpropathrin, mitochondrial dynamics, antioxidant activity, anti-inflammatory activity, antiapoptotic activity

## Abstract

**Introduction:** The synthetic pyrethroid derivative fenpropathrin (FNE), a commonly used insecticide, has been associated with various toxic effects in mammals, particularly neurotoxicity. The study addressed the hallmarks of the pathophysiology of Parkinson’s disease upon oral exposure to fenpropathrin (FNE), mainly the alteration of dopaminergic markers, oxidative stress, and molecular docking in rat models. In addition, the protective effect of curcumin-encapsulated chitosan nanoparticles (CRM-Chs-NPs) was also assessed.

**Methods:** In a 60-day trial, 40 male Sprague Dawley rats were divided into 4 groups: Control, CRM-Chs-NPs (curcumin-encapsulated chitosan nanoparticles), FNE (15 mg/kg bw), and FNE + CRM-Chs-NPs.

**Results:** FNE exposure induced reactive oxygen species generation, ATP production disruption, activation of inflammatory and apoptotic pathways, mitochondrial function and dynamics impairment, neurotransmitter level perturbation, and mitophagy promotion in rat brains. Molecular docking analysis revealed that FNE interacts with key binding sites of dopamine synthesis and transport proteins. On the other hand, CRM-Chs-NPs mitigated FNE’s toxic effects by enhancing mitochondrial dynamics, antioxidant activity, and ATP production and promoting anti-inflammatory and antiapoptotic responses.

**Conclusion:** In summary, FNE appears to induce dopaminergic degeneration through various mechanisms, and CRM-Chs-NPs emerged as a potential therapeutic intervention for protecting the nervous tissue microenvironment.

## 1 Introduction

Pyrethroids, widely used for their insecticidal properties, raised great concern due to their potential role in disrupting crucial cellular processes by impairing mitochondrial dynamics (Gajendiran and Abraham, 2018). The residues of pyrethroids can alter mitochondrial morphology, dynamics, and function, impairing energy production and cellular homeostasis (Jana et al., 2011). Additionally, exposure to these pesticides is associated with heightened oxidative stress, where an imbalance between reactive oxygen species (ROS) production and antioxidant defenses may trigger neurodegenerative phenomena (Mohamed et al., 2019). Moreover, pyrethroid-induced mitochondrial dysfunction is correlated with the accumulation of senescent mitochondria, impairing their clearance and contributing to cellular damage (Cui et al., 2010; Ryan et al., 2015). Mitochondrial dysfunction is also a major cause of neurodegenerative diseases, especially Parkinson’s disease (PD) (Moradi Vastegani et al., 2023).

Fenpropathrin (FNE), classified as an alpha-cyano pyrethroid, is known to induce neurotoxicological responses (Weiner et al., 2009). Initially, due to its rapid metabolism and clearance, FNE was perceived as relatively harmless to mammals (Proudfoot, 2005). However, it can exert prolonged effects when introduced into aquatic environments, posing a substantial threat to fish populations by accumulating within their tissues (Brander et al., 2016). Research revealed that FNE and deltamethrin can reduce kynurenic acid (KYNA) concentration in rat cortical slices (Zielinska et al., 2005), reflecting the findings in PD patients’ brain tissue and cerebrospinal fluid (Ogawa et al., 1992).

Curcumin (CRM), a bioactive compound found in the spice turmeric, has garnered significant attention for its diverse therapeutic properties. Renowned for its potent anti-inflammatory, antioxidant, and neuroprotective effects, curcumin has been extensively studied for its potential in combating various diseases and promoting overall health. Its ability to modulate multiple cellular signaling pathways makes it a promising candidate for the prevention and treatment of conditions such as cancer, neurodegenerative diseases, cardiovascular disorders, and metabolic syndrome.

Moreover, curcumin’s favorable safety profile and relatively low cost further contribute to its appeal as a natural remedy. However, challenges like poor bioavailability have prompted ongoing research to enhance its efficacy through innovative delivery systems (Bavarsad et al., 2019). Various formulations have been employed to enhance the bioavailability of curcumin, a compound with neuroprotective potential (Arya et al., 2018). These innovative approaches aim to protect curcumin from chemical degradation and include methods such as encapsulation into nanoparticles or microparticles, often integrated into food or supplements. In this sense, polysaccharide-based systems, e.g., chitosan, emerged as promising tools due to their exceptional physicochemical properties (Khater et al., 2021). Chitosan (Chs), a natural polysaccharide, possesses advantageous traits, including biocompatibility, biodegradability, and mucoadhesive capabilities (Lin et al., 2022). Chitosan-encapsulated curcumin nanoparticles (CRM-Chs-NPs) have already shown improved stability of curcumin in cervical cancer cells, bioavailability, and cellular uptake. Besides, CRM-Chs-NPs demonstrated an elevated uptake in the SiHa cells regarding free CRM (Khan et al., 2018).

Neuron impairment, followed by increased ROS production (Golpich et al., 2017), can lead to the onset and worsening of PD (Abdelnour et al., 2020; Agnihotri and Aruoma, 2020). Moreover, specific genetic mutations in genes like PTEN-induced kinase 1 (*Pink1*), *Parkin*, and protein deglycase 1 (*DJ-1*) can also play a crucial role in PD if they occur in a recessive manner (Sekine and Youle, 2018). These genes are essential for maintaining a healthy balance in the mitochondria and a process called mitophagy, which helps to remove damaged mitochondria (Burbulla et al., 2017).

Additionally, neurological degeneration and PD can also be triggered by exposure to environmental toxins like 1-methyl-4-phenyl-1,2,3,6-tetrahydropyridine (MPTP), 6-hydroxydopamine hydrobromide (6-OHDA), rotenone, and FNE (Filichia et al., 2016). These pollutants can impair mitochondria function or even disrupt them. The mitochondrial complexes I-V can be directly or indirectly affected by oxidative stress resulting from exposure to FNE, leading to energy deficits, increased ROS production, mitochondrial dysfunction, and ultimately, neuronal degeneration, a central feature of PD (Tong et al., 2018). Mitochondrial complexes can have several detrimental effects, including energy deficits via reduced ATP production from damaged complexes, ROS accumulation, and oxidative stress that further damage the complexes and exacerbate oxidative damage (Sas et al., 2007; Reddy et al., 2011).

This study aimed to understand the FNE hallmarks of the pathophysiology of PD, namely, the alteration of dopaminergic markers and oxidative stress, as well as the examination of key markers through histological and immunohistochemical staining in a rat model. Additionally, the potential benefits of CRM-Chs-NPs in counteracting the harmful effects of FNE on nerve tissue were explored. The selected dose of FNE was based on the human exposure levels as the NOAEL, identified at 300 ppm (equivalent to 15 mg/kg/bw per day), and was determined by assessing clinical indicators such as tremors, reductions in body weight, decreased blood clotting time in females, and potentially elevated alkaline phosphatase levels in both sexes at 600 ppm (equivalent to 30 mg/kg/bw per day) (Hend and Butterworth, 1976).

Additionally, the dose of CRM-Chs-NPs used in the current study was based on recent research findings, which strongly support their effectiveness and demonstrated their superior efficacy and tissue bioavailability compared to molecular curcumin in countering the harmful effects of FNE exposure on the reproductive system and hepatotoxicity (Alqahtani et al., 2023; Mohamed et al., 2023).

## 2 Materials and methods

### 2.1 Preparation and characterization of CRM-Chs nanoparticles (CRM-Chs-NPs)

CRM-Chs-NPs were prepared using the standard ionic gelation synthesis method (Kunjachan and Jose, 2010). The morphology of nanoparticles was investigated using high-resolution transmission electron microscopy (JEM-2100, JEOL, Japan, Tokyo). Zeta potential and particle size analyses were conducted using a Zeta Sizer (Nano-ZS, Malvern Instruments Ltd., Zetasizer Ver, Malvern, United Kingdom). Furthermore, the CRM-Chs-NPs surface functional groups were identified through Fourier-Transform Infrared (FTIR) spectroscopy spanning the 400–4,000 cm⁻^1^ range (ALPHA II Compact FT-IR spectrometer, Bruker, Germany). Notably, the distinctive characteristics of nanoparticles were previously elucidated and documented in a study (Alqahtani et al., 2023).

### 2.2 Animals and experimental design

A group of 40 adult male Sprague Dawley rats (10-week-old and mean weight 142 ± 0.46 g) from the Animal Housing Unit at Zagazig University’s Faculty of Veterinary Medicine were enrolled. The rats were acclimated for 12 days with a 12-h light/dark cycle with unrestricted access to food and water.

Rats were randomly assigned into 4 groups: the first group (control) received corn oil (1 mL/animal) (Arma Food Industries, Egypt), the second group (CRM-Chs-Nps) received chitosan-encapsulated curcumin in distilled water at 50 mg/kg bw (Abd-Elhakim et al., 2022), the third group (FNE) received FNE at 15 mg/kg bw (Mohamed et al., 2019), and fourth group (CRM-Chs NPs + FNE) simultaneously received the chitosan-encapsulated curcumin and FNE at the same above-cited concentrations. The corn oil vehicle was used to dissolve FNE, ensuring consistency in administration across experimental groups. In our study, distilled water was not utilized as a vehicle rather it constituted a component of the preparation of CRM-Chs-NPs, tested separately from FNE. Throughout the 60-day experiment, each group was orally given the compounds via a dedicated feeding needle.

The study was approved by the Ethics Committee at Zagazig University, Egypt (Approval number: ZU-IACUC/2/F/101/2022). Procedures were conducted according to the regulations outlined in the U.K. Animals (Scientific Procedures) Act of 1986, the EU Directive 2010/63/EU on animal experimentation, and the principles and recommendations outlined in the ARRIVE guidelines (Percie du Sert et al., 2020).

### 2.3 Chemicals

FNE (Danitol^®^) was purchased from Sumitomo Chemical Co. Ltd (Saint Didier au Mont d’Or, France), CRM from Sigma Aldrich Co. (St. Louis, Missouri, United States), and low molecular weight chitosan (MW: 100–300 KDa) from Across Co. Ltd. (Chuncheon-si, South Korea). Notably, all other compounds and reagents in this analysis exhibited a ≥99.99% purity. GSH and Caspase 3 were purchased from (Cusabio Biotech Co., Ltd.) (Beutler et al., 1963), SOD, CAT, and MDA (MyBioSource, San Diego, United States) (Spitz and Oberley, 1989), ROS (Life Sciences, Mongkok Ki, Hongkong), ATP (Assay Genie, Dublin, Ireland) (Aebi, 1984), glutamate and dopamine from Biomatik (Wilmington, Delaware, United States).

### 2.4 Collection of samples and biochemical analysis

The rats were anesthetized by isoflurane 5% inhalation via a mask and air pump (R510–25, RWD life science, San Diego, CA) for 3 min, and then were euthanized by cervical dislocation. The two primary regions of interest for dopamine, glutamate, and oxidative stress markers analysis are the striatum and the prefrontal cortex. These brain tissue regions were included for the preparation of tissue homogenate, which was placed in 20 mL of 1X PBS and stored overnight at ≤ −20 °C. Then, each sample was centrifuged at 20,000 ×*g* for 15 min. The collected supernatant was stored for the detection of oxidative stress [malondialdehyde (MDA), ROS and adenosine triphosphate (ATP) and antioxidant (superoxide dismutase (SOD), catalase (CAT) and glutathione (GSH)] markers, neurotransmitters (dopamine, glutamate) and apoptotic indices (Caspase 3) using Enzyme-linked Immunosorbent Assay (ELISA) according to the manufacturer instructions. The samples of brain hemispheres for gene expression were frozen at −80 °C. Other tissue samples were separated in a neutral buffered formaldehyde solution for histopathological and immunohistochemical staining sections. The brain was dissected into the cerebrum, including the hippocampus and cerebellum. The cerebellum was longitudinally dissected, and the hippocampus was cross-sectionally dissected at the mammillary body and tuber cinereum level, according to Treuting et al. (2017). Cerebral samples were taken from the midbrain above the hippocampus, where dopaminergic neurons are common.

### 2.5 Quantitative real-time RT-PCR (qRT-PCR)

Trizol (Invitrogen; Thermo Fisher Scientific, Waltham, MA, United States) was used for total RNA extraction from 30 mg of brain tissue. We evaluated the RNA quality to detect the A260/A280 ratio using the Nano DropVR ND-1000 Spectrophotometer (NanoDrop Technologies, Wilmington, DE, United States) for 1.5 mL of the RNA. A High-Capacity cDNA Reverse Transcription Kit cDNA Kit for cDNA synthesis (Applied Biosystems™, Waltham, MA, United States) was used. The primers were designed for the tested genes, including apoptosis genes *Casp 3, Bcl-2, Bax*, and *Sod1*), mitochondrial complex genes (*5 NADH oxidoreductase* (*Complex I*, *Succinate oxidoreductase* (*Complex II*), *Cytochrome c oxidoreductase* (*Complex III*), *Cytochrome c oxidase* (*Complex IV*), *ATP synthase F1 subunit alpha* (*ATP5F1α*), *Dynamin-related protein 1* (*Drp1*). Mitochondrial homeostasis, mitophagy-related, and mitofusins (*Mfn1* and *Mfn2*) genes were evaluated according to their manufacturer instructions (Sangon Biotech Beijing, China) as provided in Table 1 (Kimmich et al., 1975; Mihara and Uchiyama, 1978; Yuan et al., 2006).

**TABLE 1:** Primers sequences, accession number, and product size for the quantitative RT-PCR for the analyzed genes in the brain tissue.

Target gene	Primers sequences	Accession no.	pb
*Gapdh*	5′-GGC​ACA​GTC​AAG​GCT​GAG​AAT​G-3′	NM_017008.4	143
	3′-ATG​GTG​GTG​AAG​ACG​CCA​GTA-5′		
*Bax*	5′-CGA​ATT​GGC​GAT​GAA​CTG​GA-3′	NM_017059.2	109
	3′-CAA​ACA​TGT​CAG​CTG​CCA​CAC-5′		
*Bcl-2*	5′-GAC​TGA​GTA​CCT​GAA​CCG​GCA​TC-3′	NM_016993.1	135
	3′-CTG​AGC​AGC​GTC​TTC​AGA​GAC​A-5′		
*Casp 3*	5′-GAG​ACA​GAC​AGT​GGA​ACT​GAC​GAT​G-3′	NM_012922.2	147
	3′-GGC​GCA​AAG​TGA​CTG​GAT​GA-5′		
*Sod1*	5′-TTGGCCGTACTATGG TGGTC-3′	NM_017050.1	120
	3′-GGGCAATCCCAATCA CACCA-5′		
*5-Nadh oxidoreductase (Complex I)*	5′-AGA​GCC​TCA​CAG​ACA​ATG​GC-3′	NM_001005550.1	70
	3′-ATG​GCT​CCT​CTA​CTG​CCT​GA-5′		
*Succinate oxidoreductase (Complex II)*	5′-GCT​CTT​GCT​GAG​ACA​CAT​CG-3′	NM_001005534.1	191
	3′-TTG​CCA​TGG​GAA​GAG​ACC​AC-5		
*Cytochrome c oxidoreductase Complex (III)*	5′-CTC​AGC​GTG​TGG​ACT​GAA​TA-3′	NM_001013185.1	127
	3′-CCA​GGT​TCT​GCA​GGT​GAG​TT-5′		
*Cytochrome c oxidase Complex (IV)*	5′-GGA​ACC​ACA​CGC​TTT​TCC​AC-3′	NM_012812.3	71
	3′-GAG​TCT​TCA​AGG​CTG​CTC​GT-5′		
*ATP synthase F1 subunit alpha (Atpf1α) Complex V*	5′-TGC​CAT​TGA​TGG​GAA​GGG​TC-3′	NM_023093.1	97
	3′-TGG​TTC​CCG​CAC​AGA​GAT​TC-5′		
*Prkn*	5′-GAA​CTG​TGG​CTG​TGA​GTG​GA-3′	NM_020093.1	105
	3′-GGT​GTT​TCC​CAT​GAG​GTC​GT-5′		
*Mfn1*	5′-CTG​GGA​CGG​AAT​GAG​TGA​CC-3′	NM_138976.2	164
	3′-CAT​GTG​AGG​GGC​CCA​ATC​TT-5′		
*Mfn2*	5′-ACC​AGC​TAG​AAA​CGA​GAT​GTC​C-3′	NM_130894.4	109
	3′-GTG​CTT​GAG​AGG​GGA​AGC​AT-5′		
*Pink1*	5′-AGG​AAA​AGG​CCC​AGA​TGT​CG-3′	NM_001106694.1	171
	3′-CTG​TTT​GCT​GAA​CCC​AAG​GC-5′		
*Drp1*	5′-GGC​AAC​TGG​AGA​GGA​ATG​CT-3′	NM_053655.3	165
	3′-CTG​TTC​TCG​GGC​AGA​CAG​TT-5′		

Gapdh: Glyceraldehyde 3-phosphate dehydrogenase; Bax: Bcl-2-associated X protein, Bcl-2: B-cell lymphoma 2, Casp 3: Caspase 3, Sod1: Super oxide dismutase, Complex I: Nadh-ubiquinone oxidoreductase complex, Complex II: succinate dehydrogenase, Complex III: cytochrome c oxidoreductase, Complex IV: cytochrome c oxidase, Complex IV: ATP, synthase F1 subunit alpha (Atpf1α), Drp1: dynamin-related protein 1, Prkn: Parkin RBR, E3 ubiquitin protein ligase, Mfn1: Mitofusin 1, Mfn2: Mitofusin 2, Pink1: PTEN-induced kinase 1, pb: base pair.

A thorough assessment of the primer sequences was conducted. The primer sequence was validated via blast analysis NCBI, and primer details were carefully revised. The stability of the expression of different housekeeping genes, including *Gapdh, Act-b*, and *Beta-2-macroglobulin*, and their CT values using the geNorm online software (https://genorm.cmgg.be) revealed that *Gapdh* was the most stably expressed without any significant difference among experimental groups.

### 2.6 Microarchitecture examination of brain tissue

Brain samples were washed in phosphate buffer saline (pH 7.4) and immersed in 10% neutral buffered formaldehyde. The conventional paraffin embedding technique was chosen for specimens (Fischer et al., 2008).

Obtained sections were stained by Hematoxylin and Eosin (H&E). The calculation was based on semiquantitative scoring of brain necrotic lesions (Gibson-Corley et al., 2013). Scoring was applied on ten randomly chosen microscopic fields at ×400 magnification and averaged. The blinded scoring of the lesions was based on the following procedure [Score scale: 0 = normal; 1 ≤ 25%; 2 = 26–50%; 3 = 51–75%; 4 = 76–100%].

### 2.7 Immunohistochemical and immunofluorescence staining

The antibodies used in this study were the monoclonal rabbit anti-vimentin (1:300), polyclonal rabbit anti-4-hydroxynonenal (4-HNE) (1:100), and monoclonal mouse anti-Bax (1:50), which were obtained from (Abcam, Cat Ab92547, Cambridge, United Kingdom), (Abcam, Cat Ab46545, Cambridge, United Kingdom) and (Sc-7480, Santa Cruz, CA, United States), respectively. Antigen retrieval was at 10 mM citrate buffer (pH 6.0) at 105 °C for 20 min for all markers. The procedures of immunohistochemical and immunofluorescence techniques were based on Saafan et al. (2023) and Noreldin et al. (2018). The studied micrographs were obtained with a digital camera (Leica EC3, Leica, Germany). The Fiji image analyzer (National Institutes of Health, Bethesda, MD, United States) was utilized to semi-quantitatively calculate the area percentages of the various immunostaining and immunofluorescence reactions (Schindelin et al., 2012; Buckels et al., 2022). Specific brown color images representing the immunoreactions resulting from the deconvolution of ten randomly chosen images were used for analysis. The color thresholds were unified for all analyzed micrographs to compute the different immunoreaction area percentages (Vis et al., 2000).

### 2.8 Molecular docking assessment

#### 2.8.1 Instruments and tools

RCSB Protein Data Bank (RCSB PDB; https://www.rcsb.org/) and AlphaFold (https://alphafold.ebi.ac.uk/) databases, and the Molecular Operating Environment (MOE 2022.02, Chemical Computing Group, Montreal, QC, Canada) software were used for protein and ligand retrieving and molecular docking.

#### 2.8.2 Ligand preparation

Curcumin and FNE’s three-dimensional structures were obtained from the PubChem database in SDF format and opened in MOE software for energy minimization and docking with target proteins.

#### 2.8.3 Protein preparation

The three-dimensional structures of rats’ caspase 8, caspase 9, caspase 3, CAT, PINK1, NADH-ubiquinone oxidoreductase, succinate dehydrogenase complex subunit C (SDHC), coenzyme Q8A (COQ8A), ATP synthase F1 subunit alpha (ATP5F1A), protein deglycase (DJ-1), dopamine transporter (DAT), tyrosine hydroxylase proteins were retrieved from RCSB Protein Data Bank and Alpha Fold protein structure databases. Target proteins were prepared for docking using MOE software (MOE 2015.10, Chemical Computing Group, Montreal, QC, Canada) by removing water and ligand molecules in the protein structures and minimizing target protein energy.

#### 2.8.4 Molecular docking analysis and visualization

Target proteins were docked with ligands by identifying the binding site and docking with the induced fit model. Finally, the protein-ligand interactions were visualized using the same software (Vilar et al., 2008).

### 2.9 Statistical analysis

All data are expressed as mean ± SEM and were analyzed using the Prism 9.0 software from GraphPad (San Diego, CA, United States). Differences among groups for mRNA expression as well as for oxidative and antioxidant enzymes, caspase 3, and neurotransmitters were analyzed using a One-Way Analysis of Variance (ANOVA) followed by Tukey’s multiple range *post hoc* test, while for the scores, a Kruskal–Wallis test followed by Dunn’s multiple comparisons test. A *p < 0.05* was considered significant.

## 3 Results

### 3.1 Biochemical alterations due to exposure to FNE and/or CRM-Chs-NPs

#### 3.1.1 Markers of antioxidant capacity, oxidative stress, and the level of ATP

The results demonstrated significant changes in various biomarkers following exposure to FNE (Table 2).

**TABLE 2:** Effect of oral dosing of CRM-Chs-NPs on the brain oxidative and antioxidant enzymes, caspase-3, and neurotransmitters of adult Sprague Dawley rats exposed to FNE for 60 days.

Estimated parameters	Control	CRM-Chs-NPs	FNE	FNE + CRM-Chs-NPs
ROS (pg/mg)	184.4 ± 13.23	147.9 ± 3.37^£££^	610.9 ± 22.22***	347.9 ± 27.94***^,£££^
ATP (ng/mg)	23.91 ± 2.67	22.46 ± 0.4^£££^	3.200 ± 0.37***	9.515 ± 1.14***^,£^
GSH (ng/mg)	163.6 ± 3.21	182.9 ± 2.35*^,£££^	51.96 ± 2.49***	115.7 ± 7.14***^,£££^
CAT (ng/mg)	6.65 ± 0.51	8.94 ± 0.31**^,£££^	0.79 ± 0.048***	3.7 ± 0.26***^,£££^
MDA (nmol/mg)	1.23 ± 0.18	0.69 ± 0.054^£££^	9.14 ± 0.31***	3.94 ± 0.32***^,£££^
SOD (U/mg)	180.8 ± 4.29	198.4 ± 3.45^£££^	52.42 ± 3.98***^,£££^	142.8 ± 5.04***^,£££^
Caspase-3 (ng/mg)	0.76 ± 0.07	0.62 ± 0.04^£££^	7.18 ± 0.43***^,£££^	4.20 ± 0.19***^,£££^
Glutamate (ug/dL)	0.98 ± 0.09	1.05 ± 0.03^£££^	7.92 ± 0.67***	3.01 ± 0.30*^,£££^
Dopamine (DA) (ng/mg)	5.19 ± 0.24	6.29 ± 0.23^£££^	0.57 ± 0.07***	2.30 ± 0.44***^,£^

ROS: reactive oxygen species, ATP: Adenosine tri-phosphate, GSH: reduced glutathione, CAT: catalase, MDA: malondialdehyde, SOD: Superoxide dismutase. Means within the same row carrying different superscripts significantly differ at *p < 0.05 or ^£^ *p* < 0.05, **p < 0.01 or ^££^ *p* < 0.01, ***p < 0.001 or ^£££^ *p* < 0.001. Values shown are means ± SE. n = 10 group (*) is the significance vs. control while (^£^) is the difference vs. FNE, group (F_ROS_ (3, 12) = 121,7; F_ATP_ (3,12) = 46.64; F_GSH_ (3,12) = 186.6; F_CAT_ (3,12) = 117.6; F_MDA_ (3,12) = 253.5; F_SOD_ (3,12) = 236.6; F_Caspase-3_ (3,12) = 169.7; F_Glutamate_ (3,12) = 77.11; F_DA_ (3,12) = 65.78).

Specifically, exposure to FNE led to a substantial increase (*p < 0.001*) in ROS levels and MDA concentration in brain tissue, showing a 2.31-fold and 6.43-fold increase, respectively, compared to the control group. Conversely, there was a significant reduction (*p < 0.001*) in ATP levels by 86.53%, CAT activity by 88.13%, GSH content by 68.24%, and SOD activity by 71.01% compared to the control group.

Remarkably, co-treatment of CRM-Chs-NPs and FNE (FNE + CRM-Chs-NPs group) significantly decreased ROS and MDA levels in rats’ brain tissue, reducing them by 43.05% and 56.89%, respectively, compared to the FNE group (*p < 0.001*). Additionally, CRM-Chs-NPs significantly reduced oxidative stress, lowering ROS levels by 19.79% and MDA levels by 43.9% compared to the control group (*p < 0.001*).

In the CRM-Chs-NPs + FNE group, a significant enhancement in the levels of GSH, CAT, and SOD by 122.67%, 368.69%, and 172.42%, respectively, was observed compared to the FNE-exposed group (*p < 0.001*). As for ATP levels, while the FNE-exposed group experienced a significant decline (86.53%) compared to the control group (*p < 0.001*), concurrent administration of CRM-Chs-NPs with FNE significantly reversed this trend, elevating ATP content in brain tissue by 195.5% compared to the FNE group (*p < 0.05*).

Furthermore, caspase-3 protein levels in the brain tissue of the FNE-exposed group significantly increased by 842.9% compared to the control group (*p < 0.001*). However, caspase-3 was significantly reduced by 41.54% following the addition of CRM-Chs-NPs in the FNE + CRM-Chs-NPs group compared to the FNE-exposed group (*p < 0.001*).

#### 3.1.2 The levels of dopamine and glutamate in the brain tissue

A significant increase in brain tissue levels of glutamate, reaching 7.06 times the values observed in control rats, was observed in the FNE-exposed group (*p < 0.001*). Conversely, dopamine levels in the same group exhibited a significant decrease of 89.07% compared to the control values (*p < 0.001*).

In the combined treatment group (FNE + CRM-Chs-NPs), there was a significant elevation in dopamine levels, with a 3.05-fold increase and a 62.02% compared to the FNE-treated group (*p < 0.05*). This demonstrated the beneficial effect of CRM-Chs-NPs in significantly elevating dopamine levels in the brains of exposed rats to FNE for 60 days (Table 2) (*p < 0.001*).

#### 3.1.3 The expression pattern of estimated genes (apoptotic and antioxidant genes)

**FIGURE 1:**
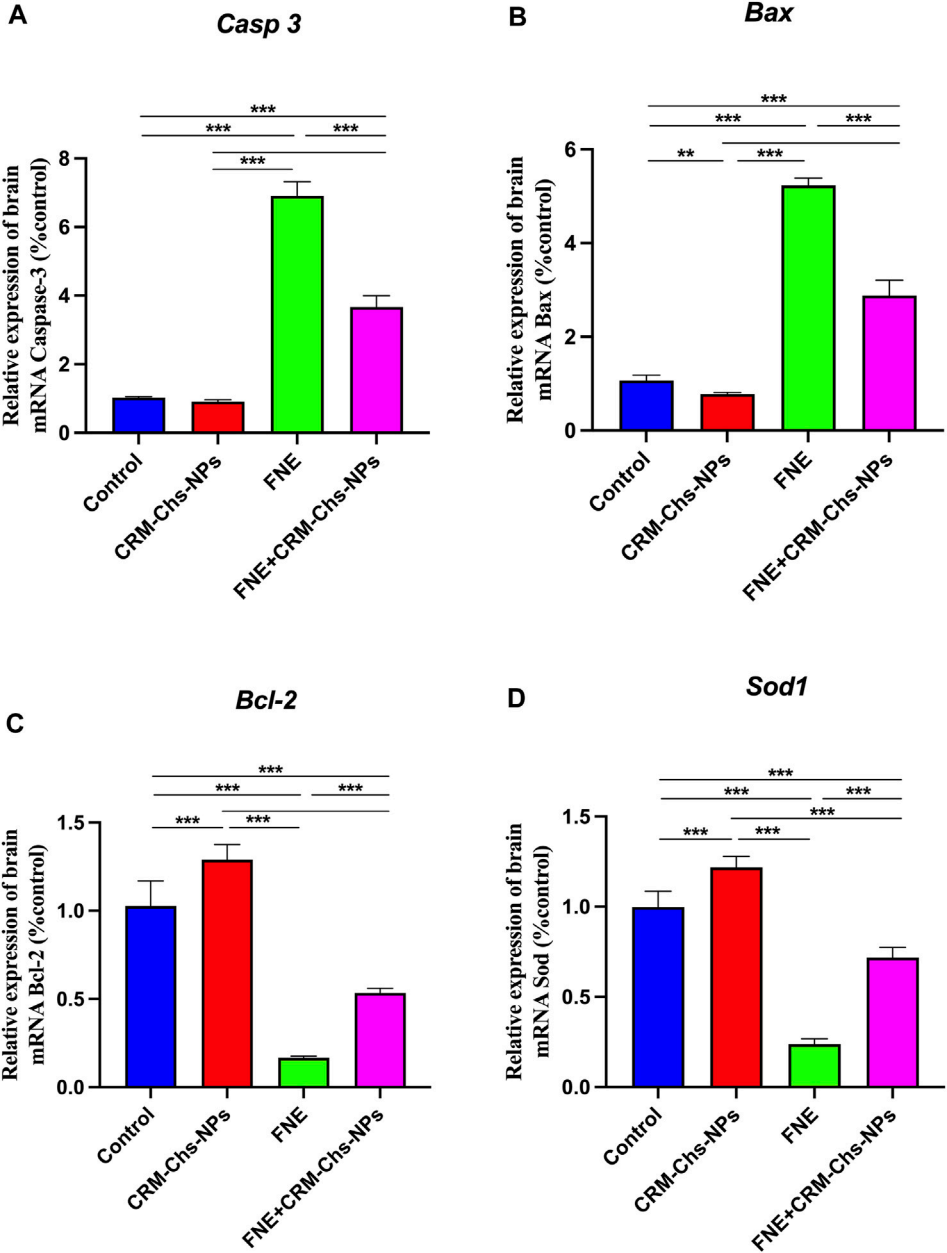
Effect of CRM-Chs-NPs oral dosing on mRNA expression of **(A)** *Casp 3*, **(B)** *Bax*, **(C)** *Bcl-2*, and **(D)** *Sod1* in the brain tissues of adult male Sprague Dawley rats exposed to FNE after 60 days. (n = 10 group), **p < 0.01, ***p < 0.001, [F_Caspase_ (3,36) = 1,154, F_BAX_ (3,36) = 1,164, F_Bcl-2_ (3,24) = 247.8, F_SOD_ (3,36) = 463.2].

As shown in Figures 1A, B, a marked upregulation of apoptosis-related genes, namely, *Casp 3* (573.03%) and *Bax* (389.52%), was observed in the brain tissues of rats exposed to FNE, indicating a significant difference (*p < 0.001*) when compared to the control group. Conversely, the expressions of *Bcl-2* and *Sod1* exhibited substantial downregulation by 83.80% and 201.79%, respectively, compared to the control group.

Notably, the group treated with CRM-Chs-NPs displayed a noteworthy decrease (*p < 0.001*) in the mRNA expressions of *Casp 3* and *Bax* (Figures 1C, D) when compared to the FNE-exposed group, with reductions of 46.88% and 44.93%, respectively.

Furthermore, in the FNE + CRM-Chs-NPs group, there was a significant (*p < 0.001*) elevation in the mRNA expressions of *Bcl-2* and *Sod1* in brain tissues, with increases of 221.27% and 201.97%, respectively, compared to the FNE-exposed group.

#### 3.1.4 Gene expression analysis of mitochondrial complex genes

**FIGURE 2:**
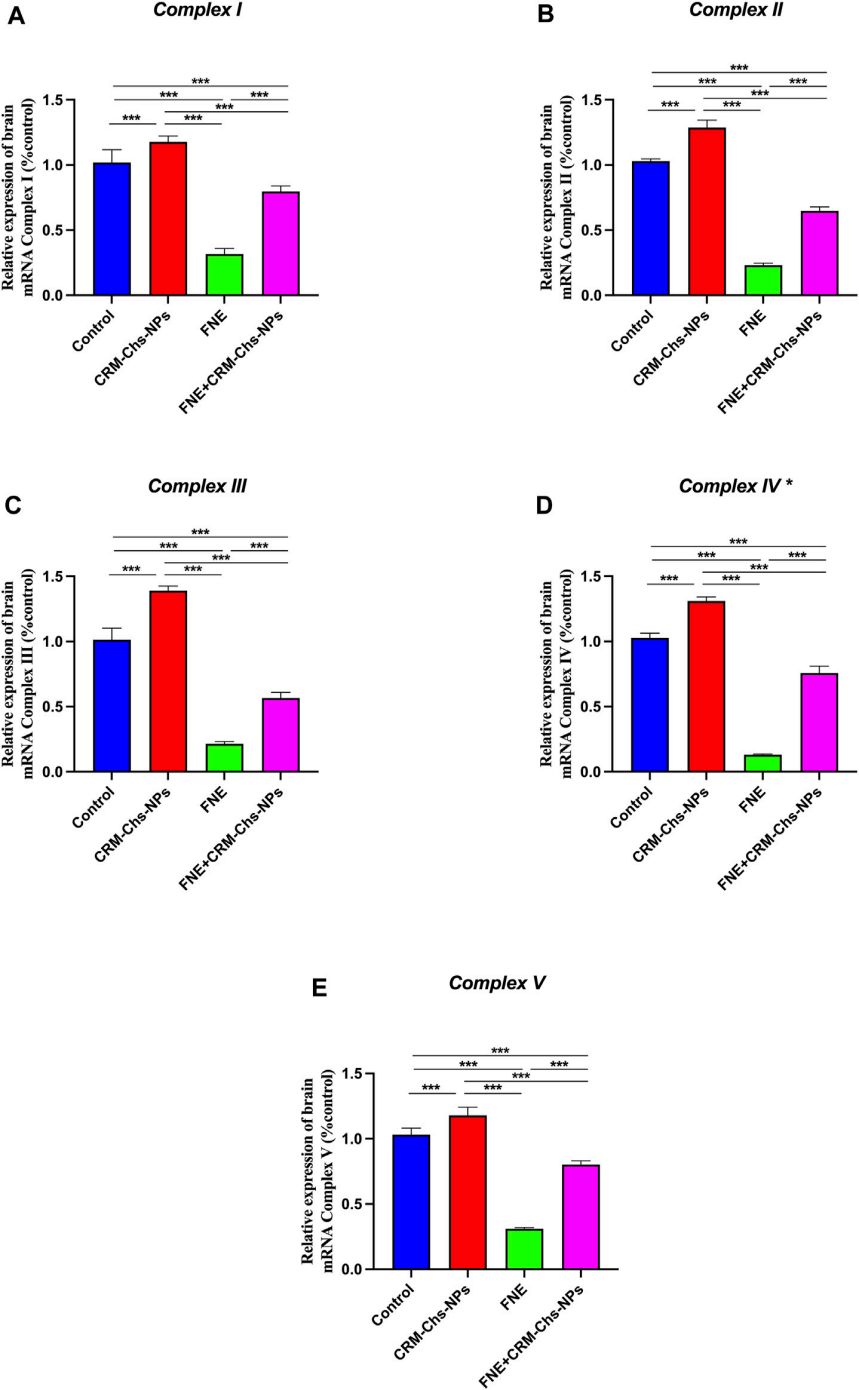
Effect of CRM-Chs-NPs oral dosing on mRNA expression of **(A)** *5-Nadh oxidoreductase* (*Complex I*), **(B)** *Succinate oxidoreductase* (*Complex II*), **(C)** *Cytochrome c oxidoreductase* (*Complex III*), and **(D)** *Cytochrome c oxidase* (*Complex IV*), **(E)** *ATP synthase F1 subunit alpha* (*Atpf1α*) (*Complex V*) in the brain tissues of adult male Sprague Dawley rats exposed to FNE for 60 days. (n = 10 group), ***p < 0.001, [F_complex I_ (3, 36) = 369.2, F_complex II_ (3, 36) = 1794, F_complex III_ (3, 36) = 956.3, F_complex IV_ (3, 36) = 2024, F_complex V_ (3, 36) = 782].

As shown in Figures 2A–E, the rats exposed to FNE exhibited a substantial downregulation of mitochondrial *Complex* genes *I, II, III, IV*, and *V* in brain tissues, with reductions of 68.98%, 77.66%, 78.81%, 87.32%, and 69.83%, respectively, compared to the control group (*p < 0.001*).

In contrast, the brain tissue of rats subjected to combined treatment with CRM-Chs-NPs and FNE showed a significant (*p < 0.001*) upregulation of the same genes, with increases of 151.83%, 181.44%, 164.95%, 458.37%, and 157.9%, respectively, compared to the FNE-exposed group. Furthermore, when assessing the comparative effectiveness of CRM-Chs-NPs and control, a statistically significant (*p < 0.001*) improvement in the gene’s activity was observed compared to the control group.

#### 3.1.5 Gene expression analysis mitophagy and mitochondrial homeostasis-related genes

**FIGURE 3:**
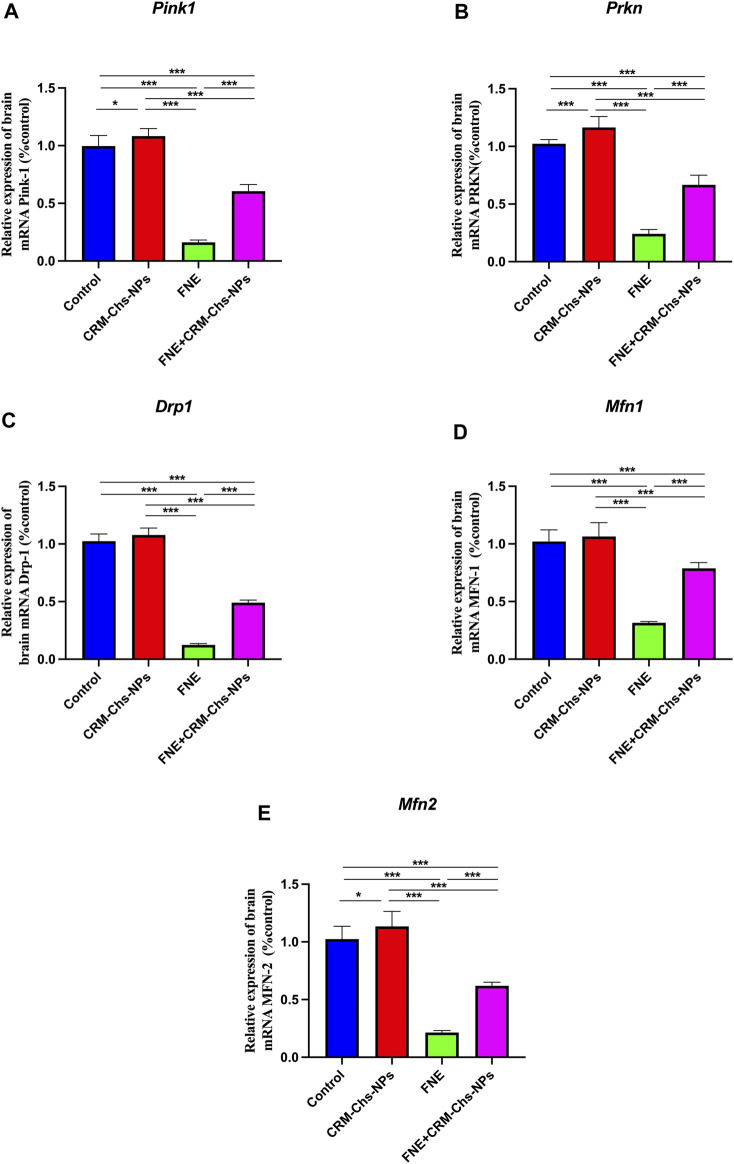
Effect of CRM-Chs-NPs oral dosing on mRNA expression of **(A)** *PTEN-induced kinase* (*Pink1*), **(B)** *Dynamin-related protein 1* (*Drp1*), **(C)** *Parkin RBR E3 ubiquitin protein ligase* (*Prkn*), **(D)** *Mitofusin 1* (*Mfn1*), and **(E)** *Mitofusin 2* (*Mfn2*) in the brain tissues of adult male Sprague Dawley rats exposed to fenpropathrin (FNE) for 60 days. (n = 10 group), *p < 0.05, ***p < 0.001, [F_Pink1_ (3, 36) = 433, F_Drp1_ (3, 36) = 1,034, F_Prkn_ (3, 36) = 357, F_Mfn1_ (3, 36) = 172.4, F_Mfn2_ (3, 36) = 230.7].

Genes associated with mitochondrial homeostasis and dynamics were also examined (Figures 3A–E).

This figure illustrates a significant downregulation (*p < 0.001*) in the expression of these genes, namely, *Pink, Drp1, Parkn, Mfn1,* and *Mfn2*, in the FNE-exposed group. The reductions were significant, 83.75%, 87.82%, 76.32%, 69.09%, and 79.08%, respectively, compared to the control group. However, when CRM-Chs-NPs were co-administered, the expression of these genes in the brain of the combined group was remarkably elevated, nearly approaching the control values compared to the FNE-exposed group. The calculated percentages are 274% in *Pink*, 293.43% in *Drp1*, 175.27% in *Parkn*, 149.84% in *Mfn1*, and 189.04% in *Mfn2* compared to the control. These findings underscore the potential of CRM-Chs-NPs to effectively mitigate the adverse effects of FNE on mitochondrial gene expression.

### 3.2 Microscopical findings

**FIGURE 4:**
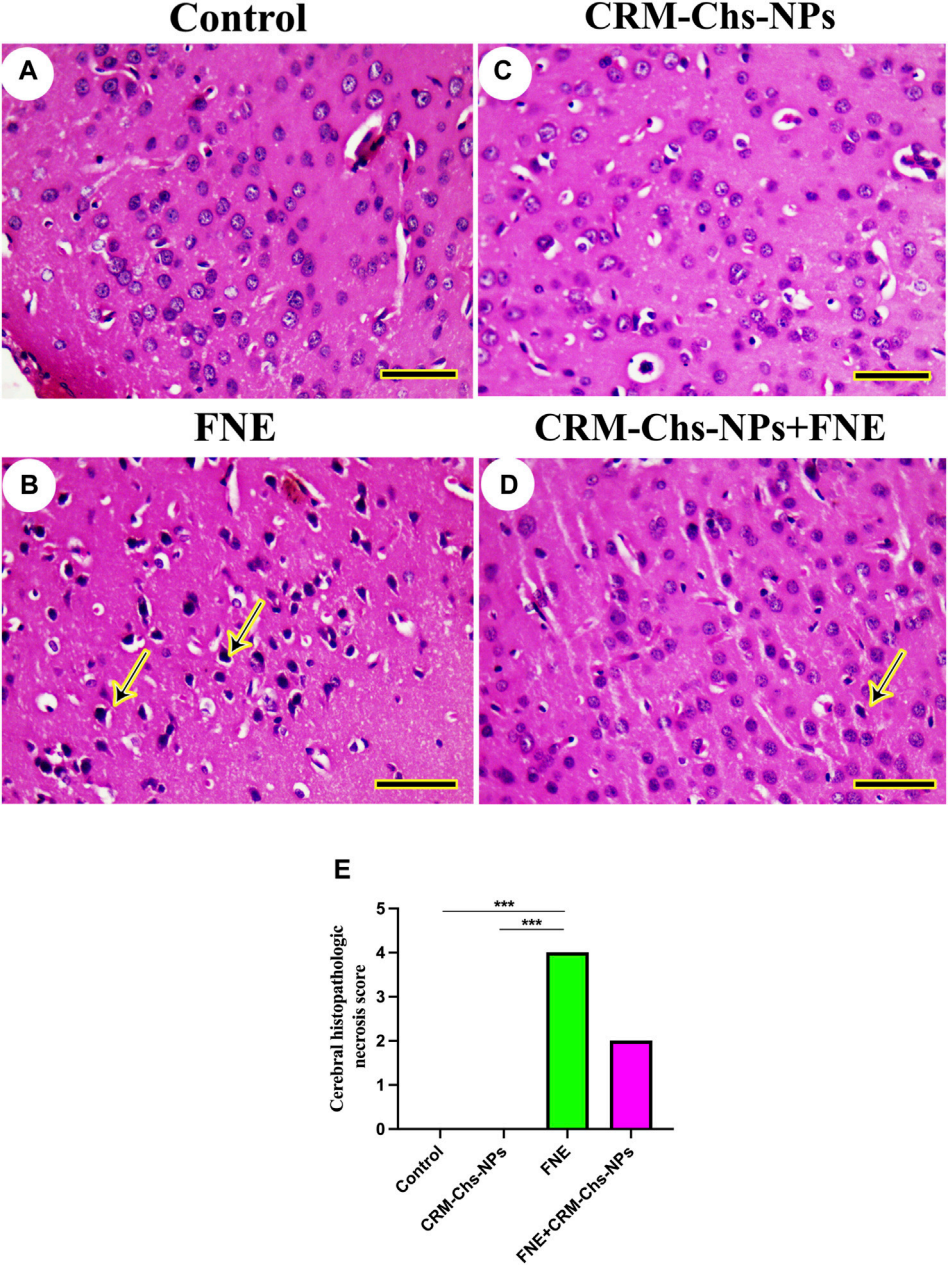
Representative photomicrograph of rat cerebrum (taken from the midbrain above the hippocampus): **(A)** control, **(B)** FNE-exposed necrotic neurons (arrow), **(C)** CRM-Chs-NPs-treated cerebrum showing normal neurons, **(D)** CRM-Chs + FNE-treated rats illustrating the improvement in cerebrum architecture with some necrotic neurons (arrow), and **(E)** graphical representation of photomicrograph score (HE, Scale bar = 50 µm). ***p < 0.001.

The study of brain histoarchitecture of negative control and CRM-Chs-NPs groups revealed normal neuropil and neurons in the brain areas, including cerebrum (taken from the midbrain above the hippocampus), cerebellum (longitudinally dissected), and hippocampus (cross-sectionally dissected at the level of the mammillary body and tuber cinereum) (Figures 4A, C).

However, we detected necrotic neurons in the FNE cerebrum (Figure 4B). CRM-Chs-NPs ameliorated the toxic effects of FNE on the cerebrum with few necrotic neurons (Figure 4D). These results were shown in the image-scoring analysis of brain histoarchitecture (Figure 4E). A significant increase in the FNE (*p < 0.001*) with respect to the control and CRM-Chs-NPs group (*p < 0.001*) was observed.

**FIGURE 5:**
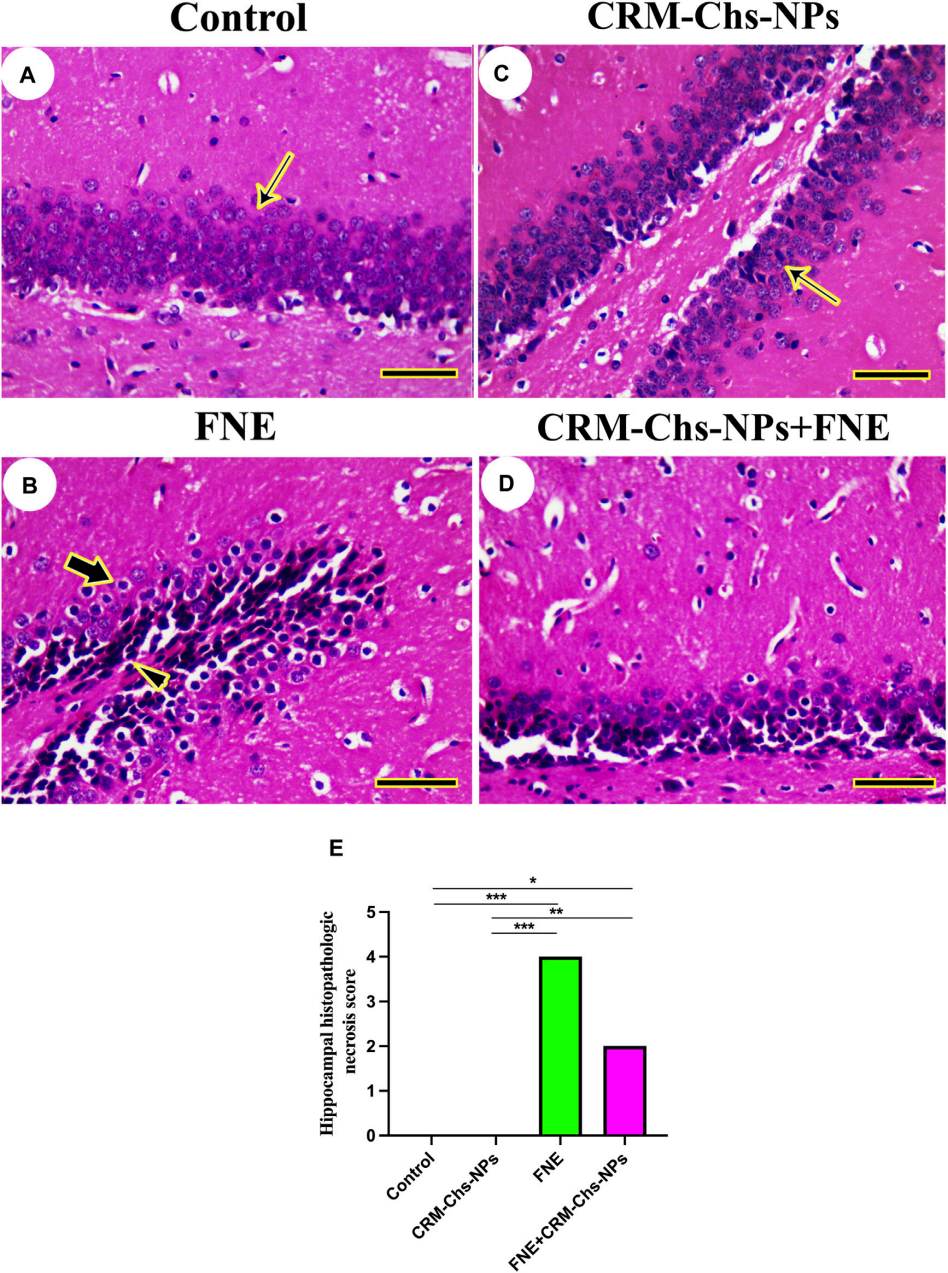
Representative photomicrograph of rat hippocampus (cross-sectionally dissected at the level of the mammillary body and tuber cinereum): **(A)** control, and **(B)** FNE reveals necrotic neurons (arrowhead) and hyperchromatic neurons (thick arrow), **(C)** CRM-Chs groups showing normal dentate gyrus (thin arrows), **(D)** CRM-Chs + FNE reveals improved hippocampal architecture, and **(E)** graphical representation of photomicrograph score (HE, *Scale bar = 50 µm*). *p < 0.05, **p < 0.01, ***p < 0.001.

The hippocampal architecture revealed typical dentate gyrus in control and CRM-Chs-NPs groups (Figures 5A, C). However, the dentate gyrus displayed necrotic neurons with a few disordered neurons in the FNE group (Figure 5B). On the other hand, CRM-Chs-NPs improved dentate gyrus structure in the CRM-Chs-NPs + FNE group (Figure 5D). These results were shown in the image-scoring analysis of hippocampal histoarchitecture (Figure 5E). A significant increase in the FNE (*p < 0.001*) and CRM-Chs-NPs + FNE (*p < 0.05*) with respect to the control and CRM-Chs-NPs group (*p < 0.001* and *p < 0.01*, respectively) was observed.

**FIGURE 6:**
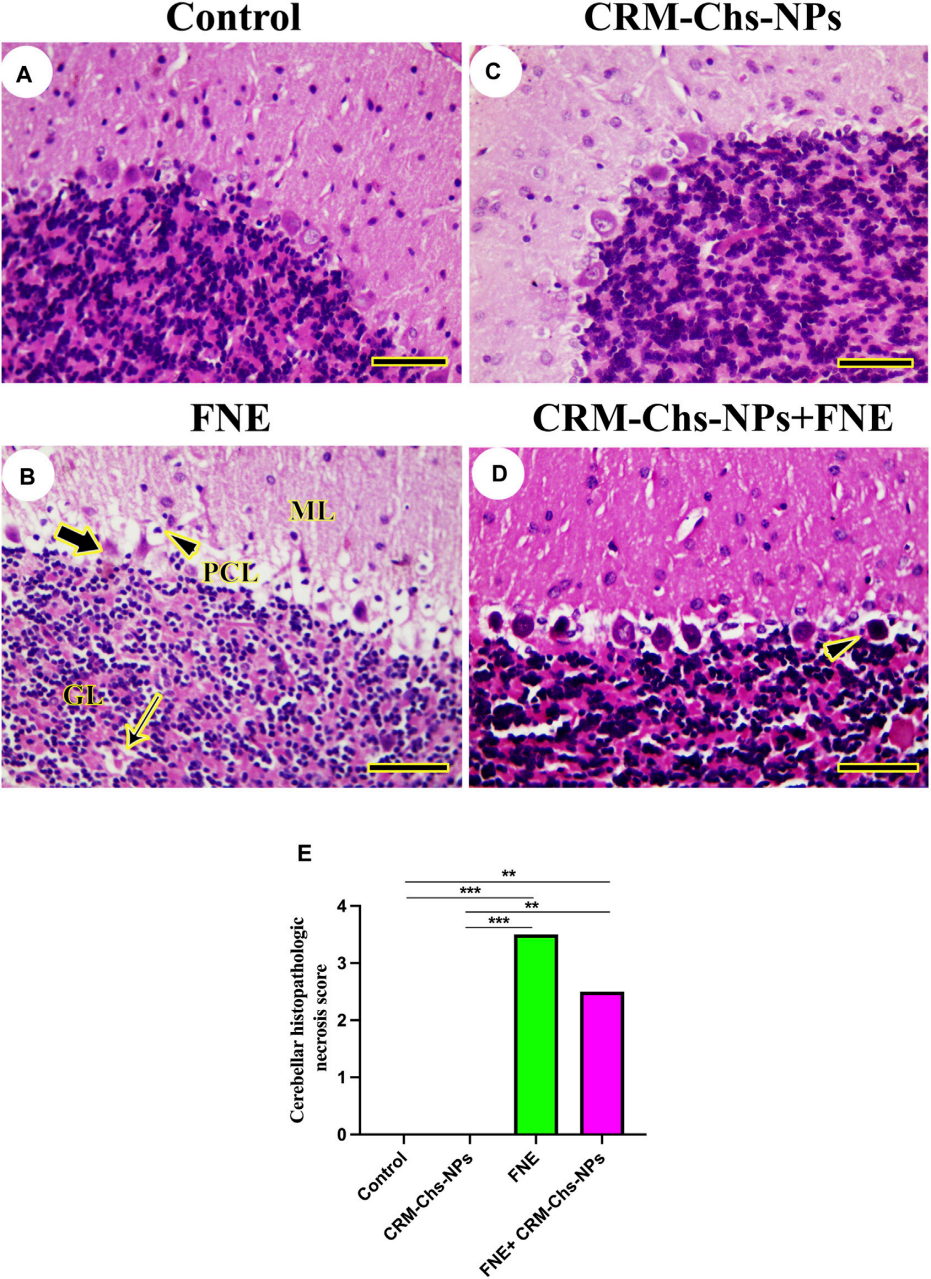
Representative photomicrograph of rat cerebellum (longitudinally dissected): **(A)** control, **(B)** FNE-treated rats revealing pyknotic Purkinje neurons (thick arrow) in the Purkinje cells layer (PCL), focal neuronal loss (thin arrow) in the granular layer (GL), and necrotic neurons (arrowhead) in the molecular layer (ML), **(C)** CRM-Chs-NPs-treated cerebellum, **(D)** CRM-Chs-NPs + FNE-treated rats showing nearly normal cerebellum with some degenerated Purkinje cells (arrowhead), and **(E)** graphical representation of photomicrograph score (HE, *Scale bar = 50 µm*). **p < 0.01, ***p < 0.001.

The investigation of cerebellar histoarchitecture of control and CRM-Chs-NPs groups revealed normal neurons (Figures 6A, C). The FNE group displayed focal cavities due to lysis or loss of granules and Purkinje neurons (Figure 6B). Few pyknotic neurons could be detected on FNE cerebellar neurons following CRM-Chs-NPs administration (Figure 6D). These results were shown in the image-scoring analysis of cerebellar histoarchitecture (Figure 6E). A significant increase in the FNE (*p < 0.001*) and CRM-Chs-NPs + FNE (*p < 0.01*) with respect to the control and CRM-Chs-NPs group (*p < 0.001* and *p < 0.01*, respectively) was observed.

### 3.3 Immunohistochemical staining

**FIGURE 7:**
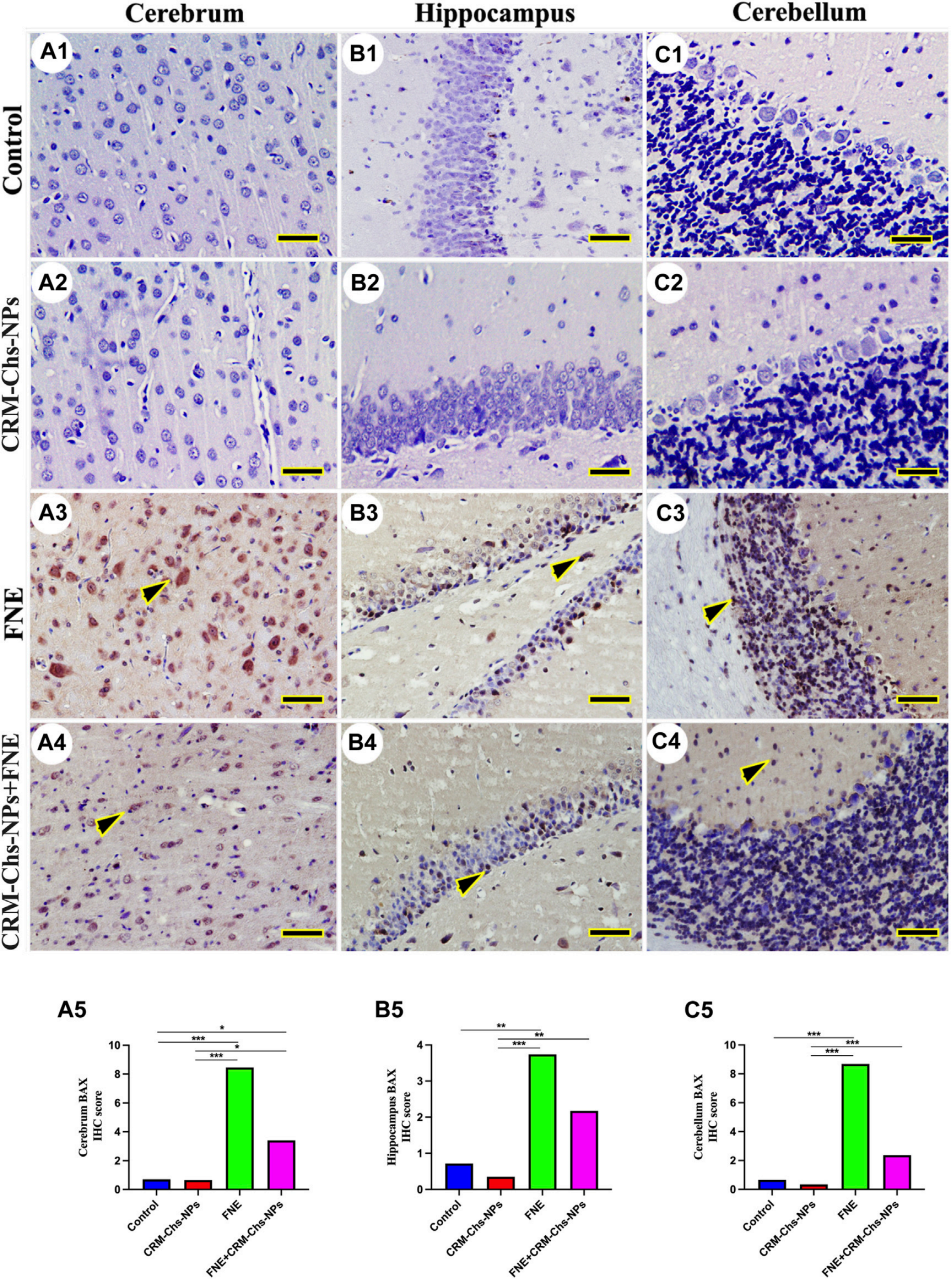
Representative photomicrograph of immunohistochemical BAX expression in the cerebrum **(A1–A5)**, hippocampus **(B1–B5)**, and cerebellum **(C1–C5)** from control **(A1,B1,C1)**, CRM-Chs-NPs **(A2,B2,C2)**, FNE **(A3,B3,C3)**, and CRM-Chs-NPs + FNE **(A4,B4,C4)** groups. Arrowheads indicate positive immune expressions (*Scale bar = 50 µm*). *p < 0.05, **p < 0.01, ***p < 0.001.

**FIGURE 8:**
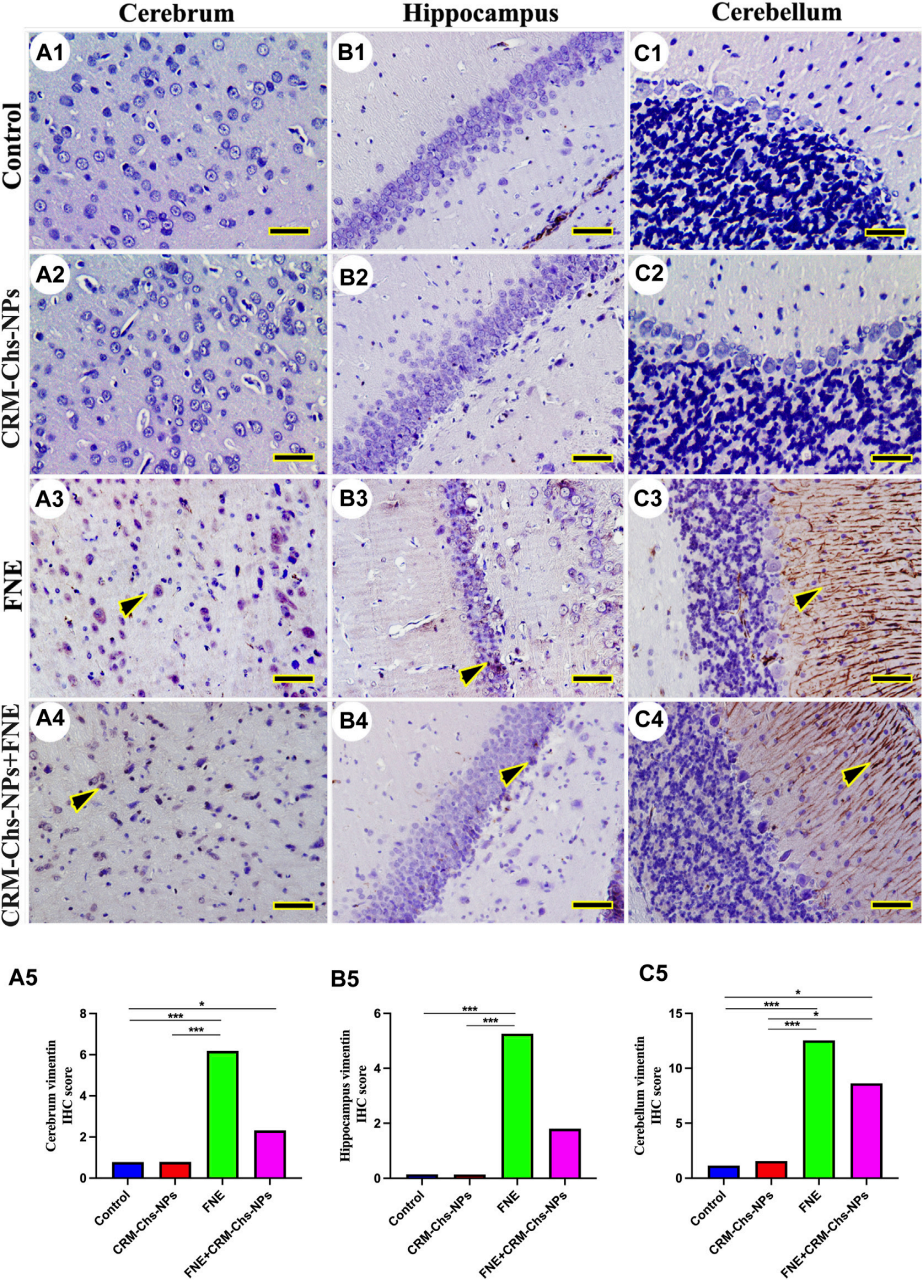
Representative photomicrograph demonstrated immunohistochemical vimentin staining in the cerebrum **(A1–A5)**, hippocampus **(B1–B5)**, and cerebellum **(C1–C5)** from negative control **(A1,B1,C1)**, CRM-Chs-NPs **(A2,B2,C2)**, FNE **(A3,B3,C3)**, and CRM-Chs-NPs + FNE **(A4,B4,C4)** groups. Arrowheads indicate positive immune expressions (*Scale bar = 50 µm*). *p < 0.05, ***p < 0.001.

There was no observable BAX immunohistochemical reaction in control and CRM-Chs-NPs groups in the cerebrum (Figures 7A1, A2), hippocampus (Figures 7B, 7B2), and cerebellum (Figures 7C1, C2), respectively. Extensive BAX reactions were detected in the nuclei of all studied brain regions of the FNE group (Figures 7A3, B3, C3). On the other hand, the co-administration of CRM-Chs-NPs to FNE decreased the BAX distribution (Figures 7A4, B4, C4). The semiquantitative analysis for the BAX area percentage revealed a significant BAX distribution in the FNE group than in control (Figure 7A5, *p < 0.001*; B5, *p < 0.01*; C5, *p < 0.001*) and CRM-Chs-NPs (Figures 7A5, B5, C5 *p < 0.001*) groups and a significant reduction in the CRM-Chs-NPs + FNE group compared to the control group, but only for the cerebrum (Figure 7A5, *p < 0.05*). A similar trend was observed for the FNE group with respect to the CRM-Chs-NPs group (Figures 8A5, B5, C5, *p < 0.001*) and for CRM-Chs-NPs + FNE compared to the CRM-Chs-NPs group (Figure 8A5, *p < 0.05*; Figure 8B5, *p < 0.01*; Figure 8C5, *p < 0.001*).

In the control and CRM-Chs-NPs groups, vimentin immunohistochemical expression showed low vimentin distribution in the cerebrum (Figures 8A1, A2), hippocampus (Figure 8B1B2), and cerebellum (Figures 8C1,C2). The FNE group showed extensive vimentin expression in all brain regions (Figures 8A3, B3, C3). CRM-Chs-NPs lowered vimentin distribution in the CRM-Chs-NPs + FNE group (Figures 8A4, B4, C4). The semiquantitative analysis for the vimentin area percentage revealed a more significant reaction in the FNE group than in control (Figures 8A5, B5, C5, *p < 0.001*), CRM-Chs-NPs (Figures 8A5, B5, C5, *p < 0.001*), and CRM-Chs-NPs + FNE group but only for the cerebrum and the cerebellum (Figures 8A5, C5, *p < 0.05*). A similar trend was observed for the FNE group with respect to the CRM-Chs-NPs group (Figures 8A5, B5, C5, *p < 0.001*) and for CRM-Chs-NPs + FNE compared to the CRM-Chs-NPs group, but only for the cerebellum (Figure 8C5, *p < 0.05*).

### 3.4 Immunofluorescence staining of 4-hydroxynonenal (4-HNE)

**FIGURE 9:**
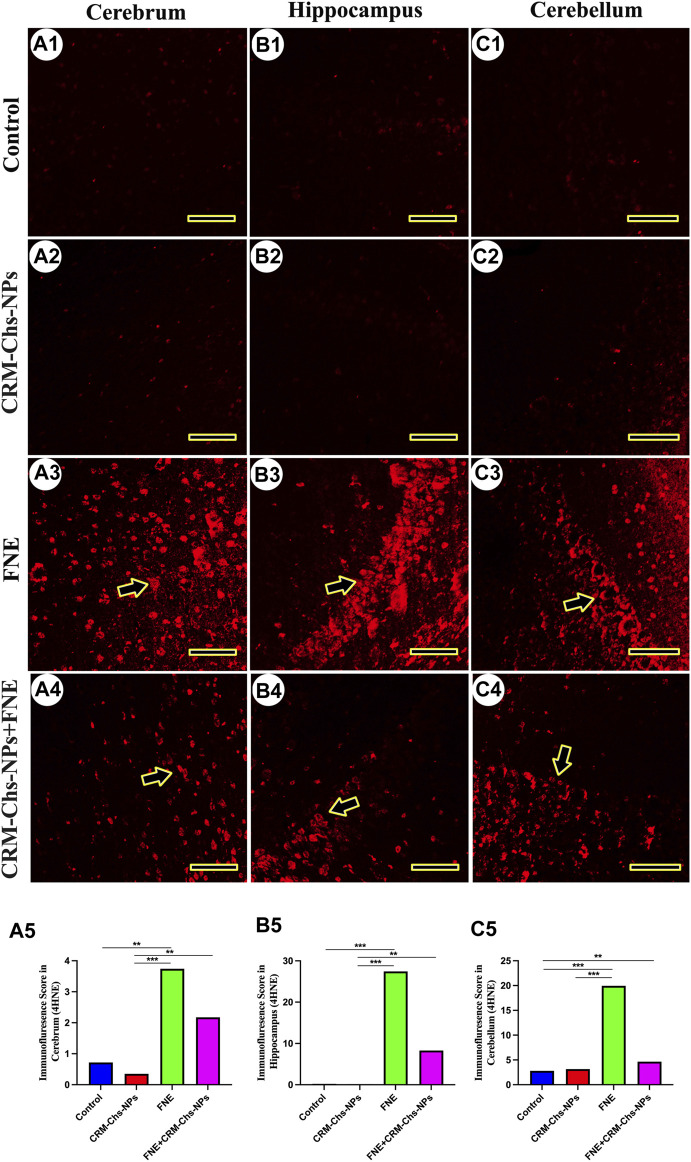
Representative photomicrograph demonstrated 4-hydroxynonenal (4-HNE) immunofluorescence in the cerebrum **(A1–A5)**, hippocampus **(B1–B5)**, and cerebellum **(C1–C5)** from negative control **(A1,B1,C1),** CRM-Chs-NPs **(A2,B2,C2)**, FNE **(A3,B3,C3)**, and CRM-Chs-NPs + FNE **(A4,B4,C4)** groups. Arrowheads indicate positive immunofluorescence expressions (*Scale bar = 50 µm*). **p < 0.01, ***p < 0.001.

The immunofluorescence of 4HNE revealed low expression in the cerebrum (Figures 9A1, A2), hippocampus (Figures 9B1, B2), and cerebellum (Figure 9C1, C2) of the control and CRM-Chs-NPs groups. Conversely, a significant 4HNE expression was detected in all brain regions of the FNE group (Figures 9A3, B3, C3), while a low expression was detected in the brain cells of the CRM-Chs-NPs + FNE group (Figures 9A4, B4, C4). The semiquantitative analysis for the 4HNE area percentage revealed a significant reaction in the FNE group than in the control (Figure 9A5, *p < 0.01*; Figure 9B5, *p < 0.001*; Figure 9C5, *p < 0.01*) and CRM-Chs-NPs + FNE groups, but only for the cerebellum (Figure 9C5, *p < 0.01*).

### 3.5 Molecular docking

**FIGURE 10:**
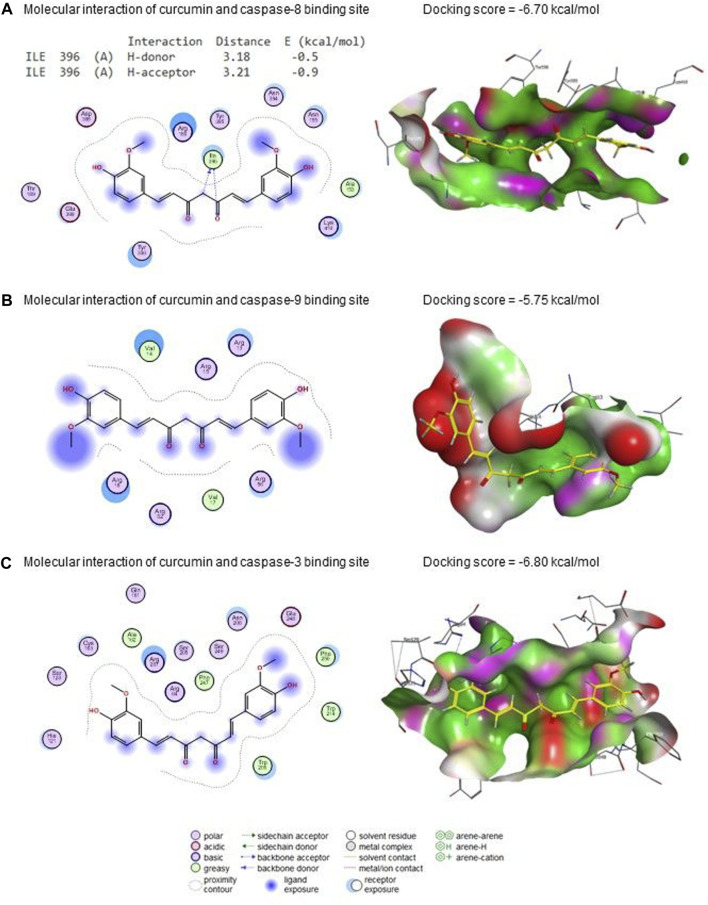
Molecular docking interaction of curcumin with **(A)** caspase 8, **(B)** caspase 9, and **(C)** caspase 3.

Figure 10 presents curcumin’s molecular interactions and docking scores with caspase-8, caspase-9, and caspase-3 binding sites. Curcumin interacted with the ILE396 (H-donor and H-acceptor) residue in caspase-8’s binding site by −6.70 kcal/mol of binding energy (Figure 10A). Also, curcumin interacted with caspase-9 (Figure 10B) and caspase-3 (Figure 10C) binding sites with energy of −5.75 and −6.80 kcal/mol, respectively.

**FIGURE 11:**
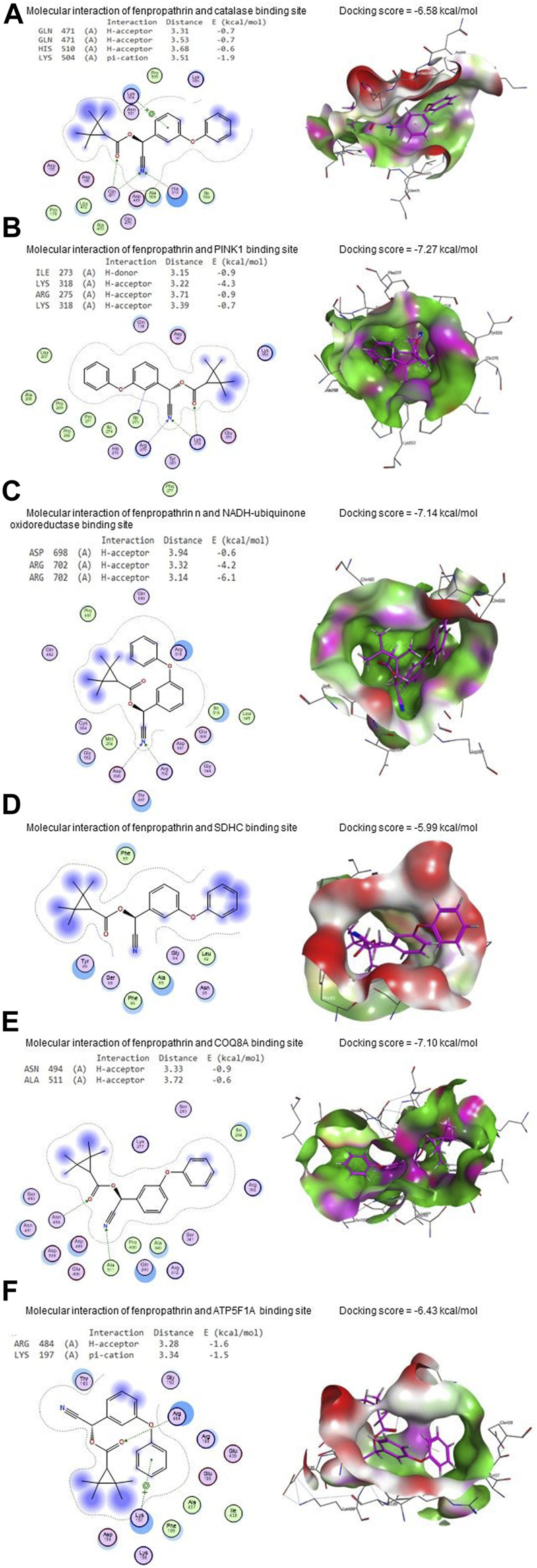
Molecular docking interaction of FNE with **(A)** CAT, **(B)** PINK1, **(C)** NADH-ubiquinone oxidoreductase, **(D)** succinate dehydrogenase complex subunit C (SDHC), **(E)** coenzyme Q8A (COQ8A), **(F)** ATP synthase F1 subunit alpha (ATP5F1A), **(G)** protein deglycase (DJ-1), **(H)** dopamine transporter (DAT), and **(I)** tyrosine hydroxylase.

FNE bound to GLN471 (two H-acceptors), HIS510 (H-acceptor), and LYS504 (pi-cation) residues in the CAT’s binding site with an energy of −6.58 kcal/mol (Figure 11A). By the energy of −7.27 kcal/mol, FNE interacted with ILE273 (H-donor), LYS318 (two H-acceptors), and ARG275 (H-acceptor) residues in the binding site of PINK1 (Figure 11B).

Regarding NADH-ubiquinone oxidoreductase, FNE bound to ASP698 (H-acceptor) and ARG702 (two H-acceptors) residues with an energy of −7.14 kcal/mol (Figure 11C). In contrast, it interacted with SDHC’s binding site with −5.99 kcal/mol of energy (Figure 11D).

As shown in Figure 11E, FNE interacted by −7.10 kcal/mol of energy with ASN494 (H-acceptor) and ALA511 (H-acceptor) residues in the binding site of COQ8A. Also, FNE is bound with ARG484 (H-acceptor) and LYS197 (pi-cation) in the binding site of ATP5F1A by an energy value of −6.43 kcal/mol (Figure 11F). By −5.73 kcal/mol of energy, FNE bound to GLU96 (H-donor), PHE119 (two H-acceptors), and GLU (pi-H) in the binding site of DJ-1, as represented in Figure 11G. The molecular interaction of the FNE and DAT binding site is represented in Figure 11H with a binding energy of −6.86 kcal/mol with ARG85 (H-acceptor) and HIS476 (H-acceptor) residues. Moreover, FNE bound to PRO327, GLY392, and SER396 residues in the binding site of tyrosine hydroxylase by an energy of −7.50 kcal/mol (Figure 11I).

## 4 Discussion

A failure in redox homeostasis is one of the typical hallmarks of PD (Fei et al., 2010). Research has shown that pyrethroids can cross the blood-brain barrier and affect the dopaminergic system, producing oxidative stress in PD (Nasuti et al., 2007). Hence, we investigated whether FNE increases brain oxidative stress in the present study. We detected ROS readily after FNE exposure in the brain tissue and found that its expression was significantly increased. In contrast, GSH, CAT, and total SOD levels as antioxidative indices were significantly reduced in the FNE group compared to the control. These results indicated that oxidation and reduction were dysfunctional following FNE exposure. Furthermore, the mRNA expression of the *Sod1* gene was significantly downregulated. These ROS molecules can disrupt mitochondrial function, impairing the electron transport chain and promoting mitochondrial dysfunction. Researchers have identified elevated free radicals and mitochondrial dysfunction as primary contributors to neuronal loss in PD brains (Nasuti et al., 2007).

Consequently, the expression of key mitochondrial complex genes, such as those involved in oxidative phosphorylation, can be altered. The current study confirmed this via the downregulated expression of mitochondrial *Complex* genes (*I-V*) in the brain tissue of the FNE group rather than the control. Other pyrethroids, including cypermethrin and deltamethrin, have also been documented to cause oxidative stress in cells (Mohamed et al., 2020; Rahman et al., 2022), thus confirming our findings.

However, treating FNE-intoxicated rats with CRM-Chs-NPs significantly decreased ROS and MDA levels while increasing SOD, CAT, and GSH contents and *Sod1* and mitochondrial *Complex* genes (*I-V*) expression in the brain tissues to alleviate oxidative stress, lipid peroxidation, and mitochondrial dysfunction. Similarly, CRM was observed to shield dopaminergic neurons from MPTP-induced neuronal damage (Stanic, 2017) and oxidative damage by restoring mitochondrial membrane potential, upregulating Cu-Zn superoxide dismutase, and inhibiting the production of intracellular ROS (Anand et al., 2007; Itokawa et al., 2008). CRM also inhibited GSH reduction in the brain by improving the GSH levels (Asai and Miyazawa, 2000) and reduced lipid peroxidation levels and protein carbonyl aggregates in the brain of the transgenic PD model of *Drosophila* (Kehlet et al., 2006). It has been proposed that the antioxidant properties of CRM and its nano form are mostly due to its ability to block ROS-generating enzymes such as microsomal monooxygenase, mitochondrial succinoxidase, and NADH oxidase (Alisi et al., 2018). In addition, CRM’s conjugated structure and enol form can boost the activity of antioxidant enzymes and scavenge free radicals (Trujillo et al., 2013). On top of that, CRM helps keep cell membranes intact by blocking peroxidation in toxicant situations (Sankar et al., 2012).

In the current study, the molecules involved in apoptosis were upregulated in the brain tissue of FNE-administered rats via detecting the levels of Caspase 3 in the brain tissue and assessing the expression of different genes related to apoptotic cascade pathway, including *Casp 3, Bax*, and *Bcl-2*. Furthermore, the immunohistochemical reactivity scoring of BAX protein was highly enhanced, while the reaction of Bcl-2 significantly declined in the brain upon exposure to FNE. These results were previously shown in some earlier studies (Mohamed et al., 2019; Mohamed et al., 2020). Reduced complex I and cytochrome P450 activity in mitochondria (Abdou et al., 2010) and mitochondrial dysfunction (Gassner et al., 1997) have been linked to pyrethroid-induced oxidative stress. Dysregulation in these genes can further exacerbate ROS production, creating a vicious cycle of oxidative stress. This cascade of events ultimately activates apoptotic pathways, initiating programmed cell death (Cao et al., 2007; Salem et al., 2023). Environmental toxicants can cause PD by disrupting mitochondrial function or dynamics (Arya et al., 2018; Khater et al., 2021; Yin et al., 2021; Lin et al., 2022) regulated by *Mfn1*, *Mfn2,* and *Drp1* genes (Gassner et al., 1997; Reddy et al., 2011). Our results indicated that FNE administration caused downregulated expression of all these proteins, thus indicating the ability of FNE to induce the pathways related to the pathophysiological changes in PD.

Similarly, dopaminergic degeneration is caused by the neurotoxin MPTP, which specifically destroys Complex I of the mitochondrial electron transport chain. This may be a similar pattern for FNE-inducing dopaminergic degeneration and then PD. Besides, in the molecular docking assessment, FNE successfully bound to the binding sites of mitochondrial Complexes (I-V), including Pink1, DJ-1, NADH-ubiquinone oxidoreductase, SDHC, COQ8A, and ATP5F1A, which is an indication for probability to occur *in vivo* leading to mitochondrial function impairment.

On the contrary, our results indicated that co-administration of CRM-Chs-NPs + FNE reduced oxidative stress (ROS and MDA) and apoptosis molecules that enhance apoptosis in the brain tissue and activate the mitochondrial complex genes. The downregulation of *Bax* and *Caspase-3* gene expression and inhibition of the *Bcl-2* gene in the brain of rats administered FNE + CRM-Chs-NPs and CRM-Chs-NPs confirmed the protective role of CRM. These improvements could help explain the key mechanisms through which CRM-Chs-NPs counteract degenerative changes caused by FNE and decrease the likelihood of PD onset. These effects could be highly correlated to what is reported in several *in vitro* and *in vivo* investigations carried out with CRM due to its antioxidant, anti-inflammatory, antiapoptotic activities, and therapeutic potential in neurodegenerative disorders (Itokawa et al., 2008). Besides, CRM-Chs-NPs potency to attenuate mitochondrial impairments by maintaining mitochondrial integrity while reducing mitochondrial oxidative stress could contribute to its neuroprotective activity (Sandhir et al., 2014). It was also previously observed that CRM could inhibit mitophagy and regulate mitochondrial dynamics in tested cells (Maiti et al., 2019). This effect could also be interrupted by the degradability of CRM-Chs-NPs, a key solution to help maintain CRM for longer periods and act on the mitochondrial homeostatic ability (Rainey et al., 2020).

Our findings also revealed a decrease in dopamine levels and an increase in glutamate levels in the brains of rats exposed to FNE for 60 days. These outcomes align with previous research, which demonstrated a connection between dopamine turnover and the acceleration of cellular dopamine uptake in rats (Husain et al., 1994). This phenomenon is associated with the upregulation of the transcription factor *Nurr1*, resulting in an increased dopamine transporter expression (Karen et al., 2001). Furthermore, Elwan et al. (2006) reported that pyrethroids, known to block sodium channels, could enhance the transcription factor *Nurr1*. This enhancement may have contributed to our study’s decrease in dopamine observed among rats exposed to FNE. It is reasonable to hypothesize that this reduction in dopamine levels may be linked to inhibited dopamine biosynthesis. This process may have occurred due to decreased synthesis of tyrosine hydroxylase and aromatic l-amino acid decarboxylase (Liu and Shi, 2006). This study also revealed a molecular interaction with average docking scores among FNE, tyrosine hydroxylase (TH), and dopamine transporter (DAT). Also, another study revealed that FNE could target the TH-positive dopamine neurons (Xiong et al., 2016). Despite this, specific staining of dopaminergic neurons and TH could explain the effect of FNE. However, it is evident from previous studies that FNE can induce PD by damaging dopaminergic neurons (Jiao et al., 2020). Thus, we focused on the toxic effects on all cerebrum, hippocampus, and cerebellum neurons and how CRM nano-formulations could ameliorate them and help prevent neurodegenerative diseases, including PD.

Besides, the reduced dopamine levels in our study, after exposure to FNE, may primarily be attributed to the loss of the biosynthesis enzyme TH. Previous research also revealed an interaction between FNE and DAT, a member of the Na^+^/Cl^−^-dependent transporter family and a key regulator of cytosolic dopamine concentration through molecular docking (Niode et al., 2021). This interaction might have contributed to the decline in dopamine levels in FNE-exposed rats, potentially leading to increased damage to dopamine neurons (Khoshbouei et al., 2003; Yoon et al., 2004). These findings underscore the consideration of FNE as a possible neurotoxin for DA and an environmental risk factor for PD. Additionally, glutamate was significantly elevated in the brains of rats exposed to FNE, as stated in many other studies (Xiong et al., 2016; Wu et al., 2023). This could be attributed to several mechanisms, among them the ability of FNE to cause glutamate buildup and exacerbation of excitotoxicity of a ubiquitin ligase that facilitates the ubiquitination and degradation of glutamate transporter 1, which causes the accumulation of glutamate in the synapse (Cao et al., 2011). Also, it was previously confirmed that several pyrethroids, including FNE, could cause efficient alterations in Ca^2+^ influx and activation of voltage-gated calcium channels, leading to the accumulation of glutamate (Cao et al., 2011; Symington et al., 2011).

Our results also revealed increased dopamine and reduced glutamate levels in the brain tissue of rats receiving CRM-Chs-NPs + FNE. This is consistent with what was found by Pan et al. (1999), who claimed the ability of CRM to increase dopamine and TH by inhibiting the glial fibrillary acidic protein (GFAP) and iNOS protein expression. Other studies also confirmed that CRM significantly protected TH-positive cells in the *substantia nigra* and restored dopamine levels in rat model (Ireson et al., 2001) and mice model (Jagatha et al., 2008). Moreover, CRM-Chs-NPs may decrease the extracellular glutamate content at a safe physiological level through a range of buffering processes, including glutamate absorption by glial cells and its conversion by glutamate decarboxylase or glutamine synthetase to the harmless glutamine (Khalil and Khedr, 2016). Furthermore, the earlier study Lin et al. (2012) demonstrated that CRM-mediated suppression of glutamate release results in downregulation of receptor expression for both metabotropic glutamate receptors 5 (mGluR5) and N-methyl-D-aspartate receptor 2B (NMDA2B).

In addition, immunohistochemistry revealed increased levels of vimentin, an intermediate filament protein of several cell types within the central nervous system (Chen et al., 2018), in the brain tissue of rats exposed to FNE compared to the control group. Vimentin also regulates the NLRP3 inflammasome since it interacts with NLRP3 in macrophages, facilitating the assembly of proteins involved in the inflammasome, including caspase-1. This latter contributes to the maturation of IL-1β (Marandi et al., 2021) and is thus associated with neuroinflammation (Khater et al., 2022). Our study also revealed an increased immunohistochemical staining of 4HNE in response to FNE exposure. This may be linked to FNE’s ability to enhance the production of ROS and induce oxidative stress, which, in turn, disrupts mitochondrial function and ATP production, leading to disrupted synaptic transmission and neuronal degeneration.

Conversely, in the CRM-Chs-NPs + FNE group, we observed a reduction in the expression of both vimentin and 4HNE in the brain tissue compared to the FNE-exposed group. This reduction could be attributed to CRM-Chs-NPs’ high ROS scavenging capacity, which promotes mitochondrial stability and ATP production (Naeimi et al., 2018). These findings provide substantial evidence for the anti-inflammatory, anti-apoptotic, and stable mitochondrial dynamics exerted by CRM-Chs-NPs.

Herein, a CRM-Chs-NPs formulation has been used to increase its bioavailability by shielding it from chemical breakdown. Such a formulation has already been used to integrate bioactive components into food or dietary supplements (McClements et al., 2009). In the current study, the oral administration of CRM-Chs-NPs to rats for 60 days resulted in no side effects. Throughout the experimental period, there were no noticeable changes in the general health state of the animals. Analysis of the recorded data indicated non-significant changes in some parameters, while in others, significant improvements were observed in the CRM-Chs-NPs group compared to the control. It is known that CRM-Chs-NPs have been extensively tested in previous studies and have been reported to be safely used in both food and drug applications. These findings reinforce the safety profile of CRM-Chs-NPs and support their potential utility in various biomedical and therapeutic applications (Jagatha et al., 2008; Rafiee et al., 2019).

## 5 Conclusion

Our findings indicate that the FNE challenge elicited signs of compromised mitochondrial function, declined ATP generation through inhibiting mitochondrial regeneration, and enhanced mitophagy. These factors collectively resulted in disrupted synaptic transmission and neuronal degeneration, outlining the harmful effects of FNE exposure on the pathophysiology of PD. However, the concurrent administration of CRM-Chs-NPs effectively reversed these detrimental effects. CRM-Chs-NPs restored ATP production, reduced proinflammatory cytokine levels, activated antioxidant enzymes, and mitigated oxidative stress. Additionally, CRM-Chs-NPs played a crucial role in regulating the activity of the mitochondrial gene complex and mitophagy, ultimately preserving the microarchitecture of brain tissue. These results underline the therapeutic potential of CRM-Chs-NPs in mitigating FNE-induced damage to dopaminergic neurons, mitochondrial health, and brain tissue microarchitectures.

## Data Availability

The original contributions presented in the study are included in the article/supplementary materials, further inquiries can be directed to the corresponding authors.
